# Defining drug/drug class refractoriness vs lines of therapy in relapsed/refractory multiple myeloma

**DOI:** 10.1038/s41408-023-00785-y

**Published:** 2023-01-11

**Authors:** Utkarsh Goel, Charalampos Charalampous, Prashant Kapoor, Moritz Binder, Francis K. Buadi, David Dingli, Angela Dispenzieri, Amie Fonder, Morie A. Gertz, Wilson I. Gonsalves, Suzanne R. Hayman, Miriam A. Hobbs, Yi L. Hwa, Taxiarchis Kourelis, Martha Q. Lacy, Nelson Leung, Yi Lin, Rahma M. Warsame, Robert A. Kyle, S. Vincent Rajkumar, Shaji K. Kumar

**Affiliations:** Division of Hematology, Mayo Clinic, Rochester, MN USA

**Keywords:** Medical research, Cancer

Dear Editor,

Prior treatments in multiple myeloma (MM) have traditionally been described using lines of therapy (LOT) [1]. A LOT is defined as one or more complete cycles of a single/combination of agents, or a planned sequential therapy consisting of several regimens [1]. The number of LOT that a patient has received does influence outcomes seen with subsequent therapies [1–3], and has been used to determine inclusion in clinical trials for relapsed/refractory multiple myeloma (RRMM) [4–9]. It has been considered as a reliable measure of prior treatments and is often used to compare results from different treatment regimens, and to guide approval of new agents for the treatment of MM. However, classification based on LOT is prone to several limitations. Although RRMM trials have considered LOT for stratifying patients [5–9], this practice assumes a uniform definition for lines of therapy [1, 10]. In reality, patients with the same number of prior lines might have received vastly different regimens, a heterogeneity that will only grow with an increasing number of available drugs. This limits the ability to compare results across different RRMM trials. Further, a LOT can change for several reasons other than disease progression—e.g., toxicity, end of planned therapy, inadequate response to therapy, etc. [1]; which are more dependent on practice patterns than disease biology. The increasing number of treatment options and different permutations and combinations of drugs makes it difficult to understand the actual significance of the number of prior lines received, owing to variability in what constitutes a line [11]. We hypothesized that a more meaningful and reliable way would be to define prior therapy by the number of drugs or the number of drug classes that a patient is refractory to, which could better reflect the disease biology.

We retrospectively reviewed RRMM patients who started a new line of systemic therapy for disease progression after January 1, 2015. Baseline characteristics collected at diagnosis included age, gender, International Staging System (ISS) stage, and interphase Fluorescence in situ hybridization (FISH) abnormalities [12]. The mSMART criteria were used for risk stratification based on FISH abnormalities [13]. We considered the relapse after January 1, 2015, for which the patients started a new line of systemic chemotherapy as the index relapse. At index relapse, we classified prior therapies by the number of LOT, as well as the number of drugs, and the number of drugs classes to which the patients were refractory (other than glucocorticoids). We defined refractoriness based on the International Myeloma Working Group (IMWG) consensus [14]. Median follow-up from diagnosis and from index relapse were calculated using the reverse Kaplan–Meier estimator method. We defined progression-free survival (PFS) from time from the start of therapy for index relapse to disease progression or death, and overall survival (OS) as time from start of therapy for index relapse to death due to all causes. Time-to-event data were analyzed using the Kaplan–Meier method, and differences between groups were assessed using the log-rank test. Cox models were used to assess the prognostic significance of various parameters in predicting PFS and OS, and Harrell’s Concordance Index (C) was used to measure the performance of different models [15].

A total of 1141 patients were included. Characteristics of the cohort, patterns of refractoriness to different combinations drugs and drug classes at index relapse, and treatments received at index relapse are described in Supplementary Tables 1 and 3. For the entire cohort, the median follow-up from diagnosis was 105 months (95% confidence interval (CI): 101–110 months), and from index relapse was 45 months (95% CI: 23–64 months). The median time from diagnosis to index relapse was 45 months (95% CI: 44–49 months). For the next line of therapy after index relapse, the PFS was 16 months (95% CI: 14–20 months), and the OS was 59 months (95% CI: 55–65 months) for the entire cohort. Overall, the median number of prior LOT was 2 (range 1–12). The median PFS for patients with 1 prior LOT (*n* = 471) was 34 months (95% CI: 29–45 months), for 2 prior LOT (*n* = 328) was 15 months (95% CI: 12–20 months), for 3 prior LOT (*n* = 167) was 11 months (95% CI: 8–13 months), and for > =4 prior LOT (*n* = 175) was 6 months (95% CI: 5–7 months) (Fig. 1A). The PFS for 1/2/3/>=4 LOT groups were significantly different from each other (*P* < 0.01 for all pairs). The median OS for patients with 1 prior LOT was 93 months, for 2 prior LOT was 60 months (95% CI: 49–NR (not reached)), for 3 prior LOT was 46 months (95% CI: 39–66 months), and for > =4 prior LOT was 30 months (95% CI: 25–41 months) (Fig. 1B). The median OS was significantly different among all pairs (*P* = 0.035 for 2 lines vs 3 lines, *P* < 0.02 for all other pairs).

**Fig. 1:**
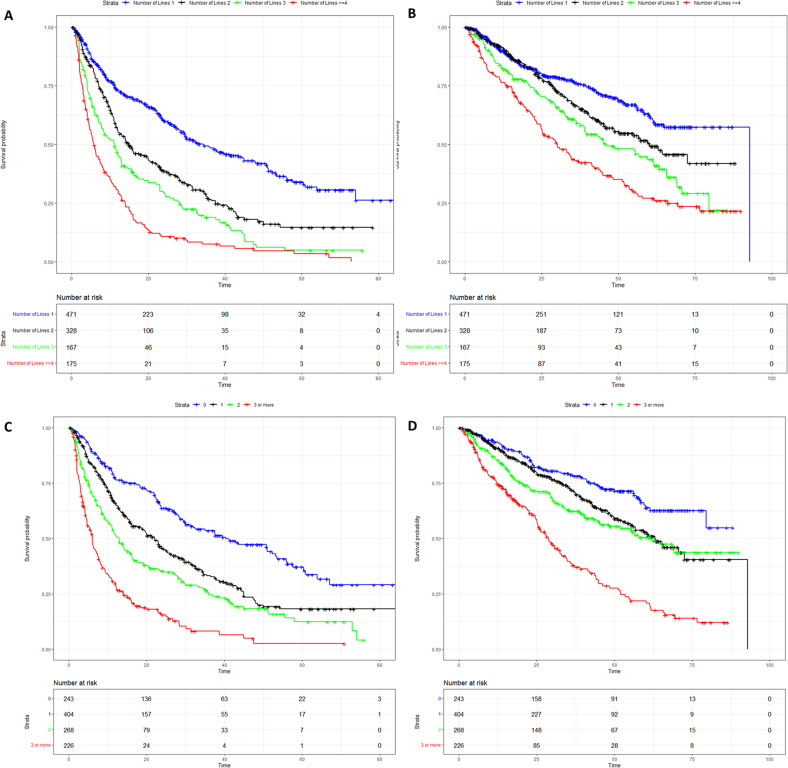
Survival curves according to different classifications. **A** Progression-free survival (PFS) by the number of prior lines of therapy. **B** Overall survival (OS) by the number of prior lines of therapy. **C** Progression-free survival (PFS) by refractoriness to the number of drug classes. **D** Overall survival (OS) by refractoriness to the number of drug classes.

The median PFS for not refractory patients was 40 months (95% CI: 30–53 months), for 1 drug refractory was 23 months (95% CI: 19–26 months), for 2 drugs refractory was 14 months (95% CI: 12–20 months), and for > =3 drugs refractory was 6 months (95% CI: 5–7 months). The PFS for all these groups were significantly different from each other (*P* = 0.016 for 1 drug refractory vs 2 drugs refractory, *P* < 0.001 for all other pairs). The median OS for not refractory patients was not reached, for 1 drug refractory was 65 months, for 2 drugs refractory was 65 months, and for > =3 drugs refractory was 30 months. The OS was not different between 1 drug refractory and 2 drugs refractory patients (*P* = 0.51) and was statistically different among other groups (*P* = 0.01 for 1 drug refractory vs not refractory patients, *P* < 0.01 for all other pairs) (Supplementary Fig. 2).

The median PFS for not refractory patients was 40 months (95% CI: 30–53 months), for 1 class refractory was 22 months (95% CI: 18–25 months), for 2 class refractory was 13 months (95% CI: 11–15 months), and for > =3 class refractory was 6 months (95% CI: 5–7 months) (Fig. 1C). The PFS for all these groups were significantly different from each other (*P* < 0.001 for all pairs). The median OS for not refractory patients was not reached, for 1 class refractory was 63 months, for 2 class refractory was 62 months, and for > =3 class refractory was 29 months (Fig. 1D). The OS was not statistically different between 1 class refractory and 2 class refractory patients (*P* = 0.14) and was statistically different among other groups (*P* = 0.001 for 1 class refractory vs not refractory patients, *P* < 0.001 for all other pairs). Multivariable analyses are described in Supplementary Figs. 3–8. Reclassification of patients from the number of prior lines of therapy to the number of drugs/ drugs classes refractory is shown in Supplementary Fig. 9.

Stratification based on drug/drug class refractoriness led to 4 well-separated group of patients, and a better separation of survival curves, vs LOT (Table 1). Harrell’s C indices for the different classification systems are described in Supplementary Table 4. Since most RRMM trials included patients with 1–3 prior lines of therapy, we evaluated the impact of classifying based on refractoriness in this subgroup of our cohort (*n* = 966). Again, classification based on drug class refractoriness led to separation into well-discriminated groups (Supplementary Fig. 10).

**Table 1:** Classification based on refractoriness to the number of drugs/drug classes vs lines of therapy.

Number of lines	Median PFS (months)	Number of drugs/drug classes refractory	PFS (number of drugs refractory to)	PFS (number of drug classes refractory to)
1	34.2	0	40.3	40.3
2	14.9	1	22.8	21.9
3	11.0	2	14.1	12.9
>=4	5.7	>=3	6.0	6.0
**Overall survival**				
**Number of lines**	**Median OS (months)**	**Number of drugs/drug classes refractory**	**OS (number of drugs refractory to)**	**OS (number of drug classes refractory to)**
1	92.8	0	NR	NR
2	60.2	1	65.0	63.1
3	46.1	2	64.4	61.8
>=4	30.2	>=3	29.5	28.7

*PFS* progression-free survival, *OS* overall survival, NR not reached.

RRMM patients are often grouped based on the number of prior lines into 1–3 prior lines and >3 prior lines, for inclusion in clinical trials [11]. However, the adoption of newer therapies in earlier lines can present a challenge to this lines-based approach. In patients who had received 1–3 prior lines of therapy in our study, we demonstrated that this seemingly homogenous cohort was also demarcated into four distinct groups, when re-classified using refractoriness. This suggests that number of drugs/ drug classes refractory can provide a better selection of a homogenous patient population for clinical trials, than what has been the case using LOT. Our study is prone to inherent limitations due to its retrospective nature and data being from a single institution. Our classification system gives equal weight to refractoriness to different drugs and refractoriness to different doses of the same drug. Finally, classification based on refractoriness does not consider the possibility of loss of resistance to drugs, or differential resistance to drugs that are a part of a combination regimen. Criteria for drug approvals still consider number of prior LOT as one of the factors. However, refractoriness to drugs/drug classes is a more consistent/ scientific definition of prior therapies as compared to prior lines. We suggest that possibly, importance only be given to refractoriness to number of drugs/ drug classes, with additional consideration to refractoriness to specific drug classes, to guide eligibility for newer MM therapies. Alternatively, a composite score incorporating both number of LOT and refractoriness could be considered in future studies. Classification based on drug/ drug class refractoriness is a robust system and has implications in patient selection for clinical trials, and in determining approval and eligibility for new therapies for RRMM in lieu of LOT.

## Supplementary information

Supplemental Material

## Data Availability

The datasets generated during and/or analyzed during this study are available from the corresponding author on reasonable request.
