# Supplementary material for: Defining drug/drug class refractoriness vs lines of therapy in relapsed/refractory multiple myeloma
Source: Blood Cancer J. 2023 Jan 11;13(1):11. doi: 10.1038/s41408-023-00785-y (PMC9834217; doi:10.1038/s41408-023-00785-y)
Supplement: Supplementary file 1 — Supplemental Material [file 41408_2023_785_MOESM1_ESM.docx]

**Supplementary material**

**Contents:**

1. **Supplementary Table 1- Baseline patient characteristics at diagnosis**
2. **Supplementary Table 2- Refractoriness to different drug classes at index relapse**
3. **Supplementary Table 3- First salvage regimen received at index relapse**
4. **Supplementary Table 4- Harrell’s C (95% CI) for the different classification systems**
5. **Supplementary Figure 1- Study flowchart**
6. **Supplementary Figure 2A- Progression Free Survival (PFS) by refractoriness to number of drugs, Figure 2B- Overall Survival (OS) by refractoriness to number of drugs**
7. **Supplementary Figure 3: Cox model hazard ratios for progression free survival (by lines of therapy)**
8. **Supplementary Figure 4- Cox model hazard ratios for overall survival (by lines of therapy)**
9. **Supplementary Figure 5- Cox model hazard ratios for progression free survival (by refractoriness to number of drugs)**
10. **Supplementary Figure 6- Cox model hazard ratios for overall survival (by refractoriness to number of drugs)**
11. **Supplementary Figure 7- Cox model hazard ratios for progression free survival (by refractoriness to number of drug classes)**
12. **Supplementary Figure 8- Cox model hazard ratios for overall survival (by refractoriness to number of drug classes)**
13. **Supplementary Figure 9A- Redistribution from lines of therapy to number of drugs refractory, Supplementary Figure 9B- Redistribution from lines of therapy to number of drug classes refractory.**
14. **Supplementary Figure 10A- Progression Free Survival (PFS) by refractoriness to number of drug classes (in patients that had received 1-3 prior lines of therapy) Supplementary Figure 10B- Overall Survival (OS) by refractoriness to number of drug classes (in patients that had received 1-3 prior lines of therapy).**
15. **Supplementary Figure 11A- Progression free survival by refractoriness to last line of therapy, Supplementary Figure 11B- Overall survival by refractoriness to last line.**
16. **Supplementary Figure 12A- Progression free survival by receipt of previously refractory to drug at index relapse, Supplementary Figure 12B- Overall survival by receipt of previously refractory to drug at index relapse.**

**Supplementary Table 1- Baseline patient characteristics at diagnosis**

| **Parameters** | **All patients (N=1141)** | |
| --- | --- | --- |
| **Demographics** | **n** | **%** |
| Age, median (IQR) | 62 years (55 – 68 years) | |
| Gender: Female | 467 | 41% |
| **Disease characteristics** |  |  |
| **ISS stage** |  |  |
| I | 291 | 26% |
| II | 326 | 29% |
| III | 254 | 22% |
| Not available | 270 | 23% |
| **FISH at diagnosis** |  |  |
| Standard risk | 589 | 52% |
| High risk | 360 | 32% |
| t(4;14) | 100 | 9% |
| t(14;16) | 20 | 2% |
| t(14;20) | 5 | 0.5% |
| del(17p) | 121 | 11% |
| gain(1q) | 184 | 16% |
| Not available | 192 | 17% |
| **M-protein isotype** |  |  |
| IgG | 635 | 56% |
| IgA | 221 | 19% |
| Light chain only disease | 210 | 18% |
| Other | 29 | 3% |
| Not available | 46 | 4% |

IQR indicates interquartile range; ISS, International Staging System; FISH, Fluorescence in situ hybridization; t, translocation; del, deletion. Percentages are rounded off.

**Supplementary Table 2- Refractoriness to different drug classes at index relapse**

| **Drug class** | **n** | **%** |
| --- | --- | --- |
| **Proteasome inhibitors** |  |  |
| Bortezomib | 408 | 36% |
| Carfilzomib | 45 | 4% |
| Ixazomib | 76 | 7% |
| Bortezomib and carfilzomib | 50 | 5% |
| Bortezomib and ixazomib | 24 | 2% |
| Carfilzomib and ixazomib | 4 | <1% |
| Bortezomib, carfilzomib and ixazomib | 4 | <1% |
| **Immunomodulatory drugs** |  |  |
| Lenalidomide | 486 | 43% |
| Pomalidomide | 60 | 5% |
| Thalidomide | 7 | ~1% |
| Lenalidomide and pomalidomide | 102 | 9% |
| Lenalidomide and thalidomide | 7 | ~1% |
| Pomalidomide and thalidomide | 1 | <1% |
| Lenalidomide, pomalidomide and thalidomide | 2 | <1% |
| **Anti-CD38 monoclonal antibodies** |  |  |
| Daratumumab | 128 | 11% |
| **Anti-SLAMF7 monoclonal antibody** |  |  |
| Elotuzumab | 13 | ~1% |
| **Alkylating agents** |  |  |
| Cyclophosphamide | 144 | 13% |
| Cisplatin | 1 | <1% |
| **Nuclear export inhibitor** |  |  |
| Selinexor | 4 | <1% |
| **Histone deacetylase inhibitor** |  |  |
| Panobinostat | 4 | <1% |
| **BCL-2 Inhibitor** |  |  |
| Venetoclax | 8 | ~1% |
| **Bendamustine** | 13 | ~1% |
| **Other classes (etoposide, doxorubicin, dinaciclib, vincristine, sorafenib, pembrolizumab, and other investigational drugs)** | 18 | ~2% |

Percentages are rounded off.

**Supplementary Table 3- First salvage regimen received at index relapse**

| **Therapy** | **n** | **%** |
| --- | --- | --- |
| PI + IMiD (without mAb or venetoclax) | 205 | 18% |
| PI (without IMiD, mAb, or venetoclax) | 145 | 13% |
| IMiD (without PI, mAb, venetoclax, or selinexor) | 140 | 12% |
| mAb (including combination with PI, IMiD, but not venetoclax) | 575 | 50% |
| Alkylator combinations (without PI, IMiD, mAb, or venetoclax) | 6 | ~1% |
| Venetoclax based (including combination with PI, IMiD, mAb) | 13 | ~1% |
| VDT PACE like regimens | 39 | 3% |
| Selinexor based combinations (with IMiD or dexamethasone) | 6 | ~1% |
| Miscellaneous (belantamab mafodotin, ASCT only, and other investigational drugs) | 12 | ~1% |

PI indicates proteasome inhibitor; IMiD, immunomodulatory drug; mAb, monoclonal antibody; VDT PACE, bortezomib, dexamethasone, thalidomide, cisplatin, doxorubicin, cyclophosphamide, and etoposide; ASCT, autologous stem cell transplantation. Percentages are rounded off.

**Supplementary Table 4- Harrell’s C (95% CI) for the different classification systems**

| **Method** | **PFS** | **OS** |
| --- | --- | --- |
| **Number of lines** | 0.642 (0.620 – 0.663) | 0.601 (0.571 - 0.630) |
| **Number of drugs refractory to** | 0.653 (0.633 – 0.672) | 0.623 (0.595 -0.650) |
| **Number. of drug classes refractory to** | 0.649 (0.629 – 0.668) | 0.621 (0.593 – 0.648) |

PFS indicates progression free survival; OS, overall survival; CI, confidence interval.

**
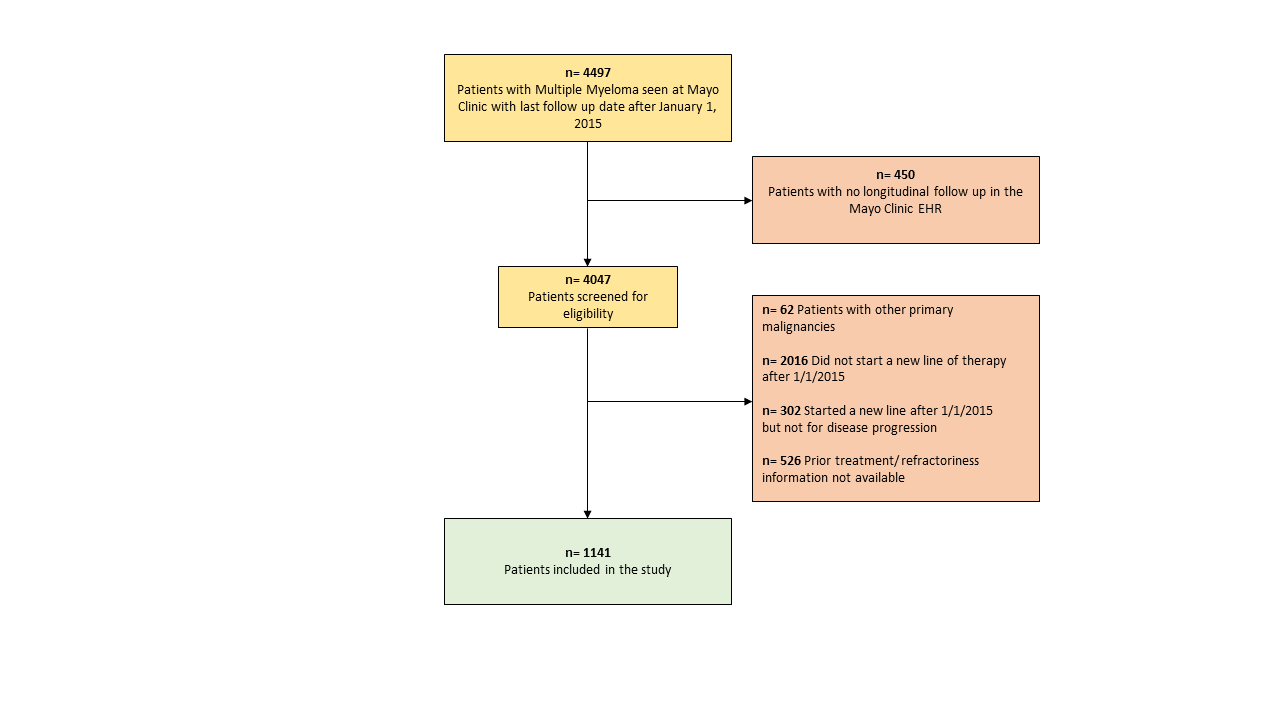
Supplementary Figure 1- Study flowchart**

**Supplementary Figure 2A- Progression Free Survival (PFS) by refractoriness to number of drugs, Figure 2B- Overall Survival (OS) by refractoriness to number of drugs**

**
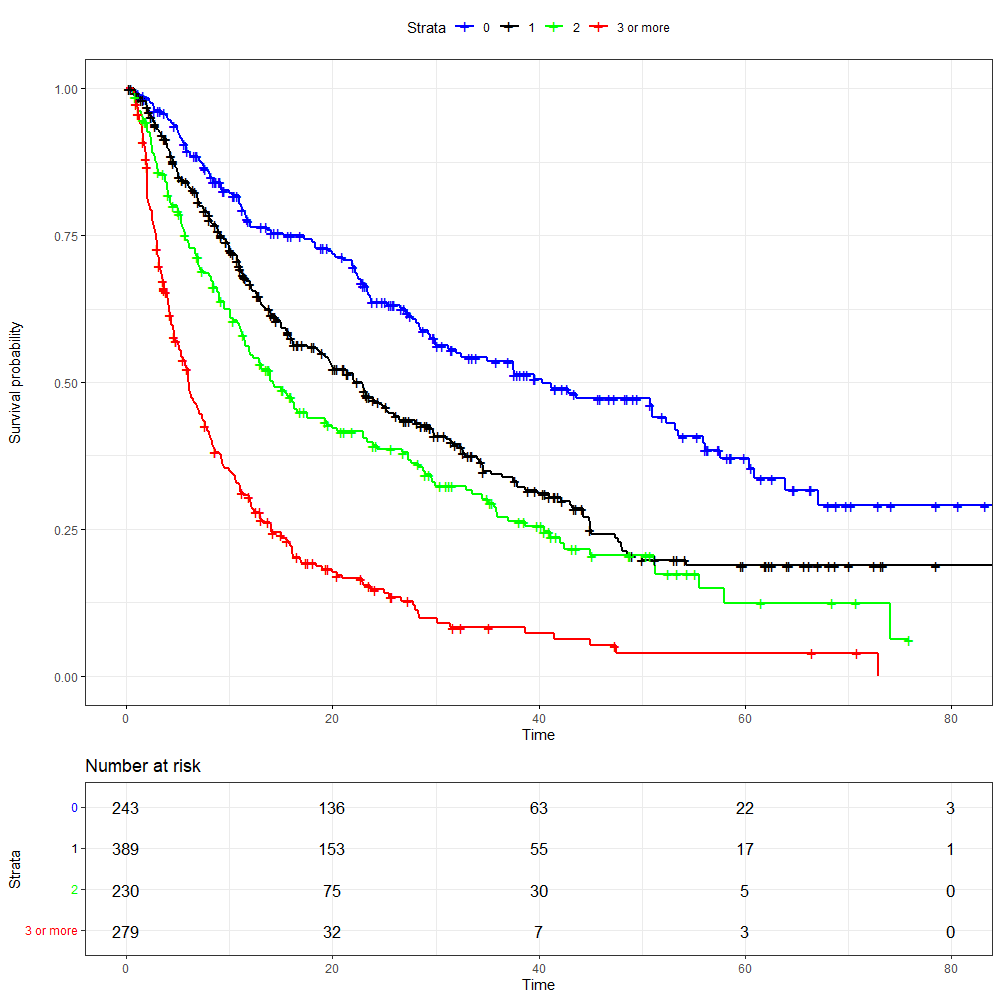

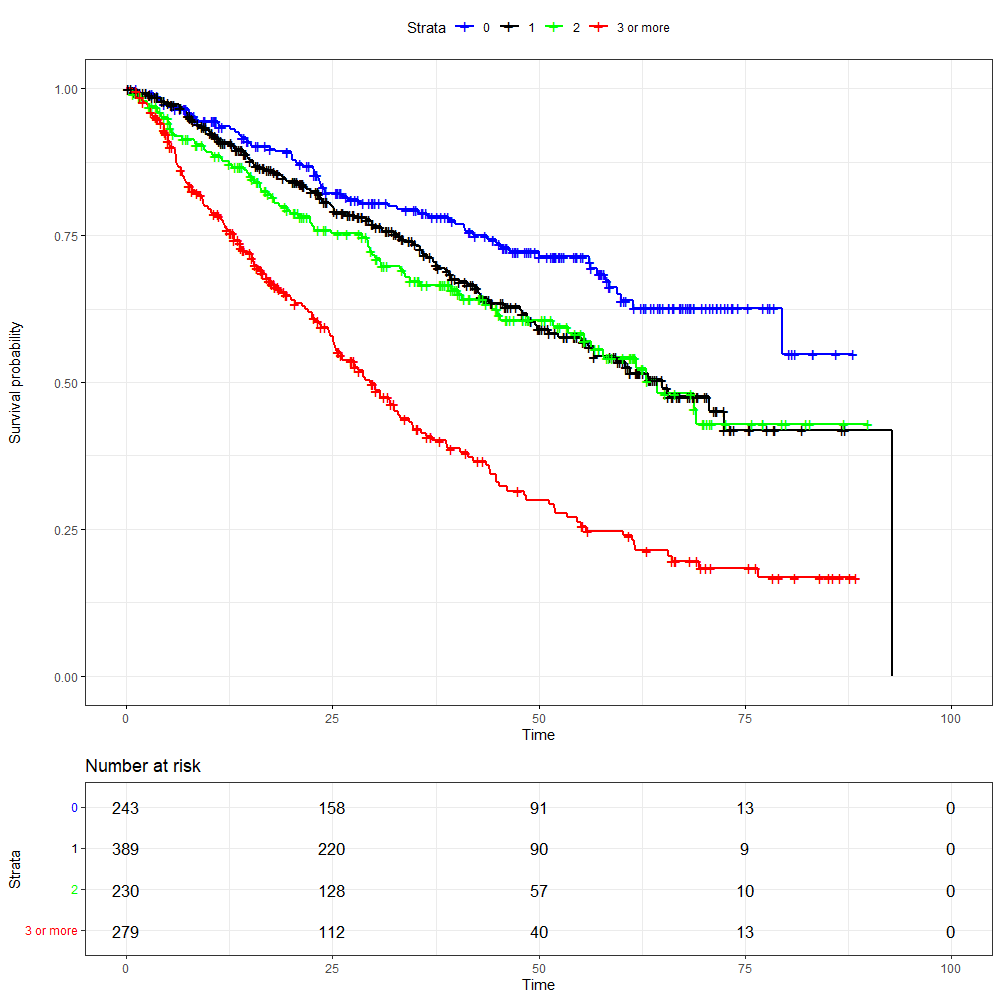
**


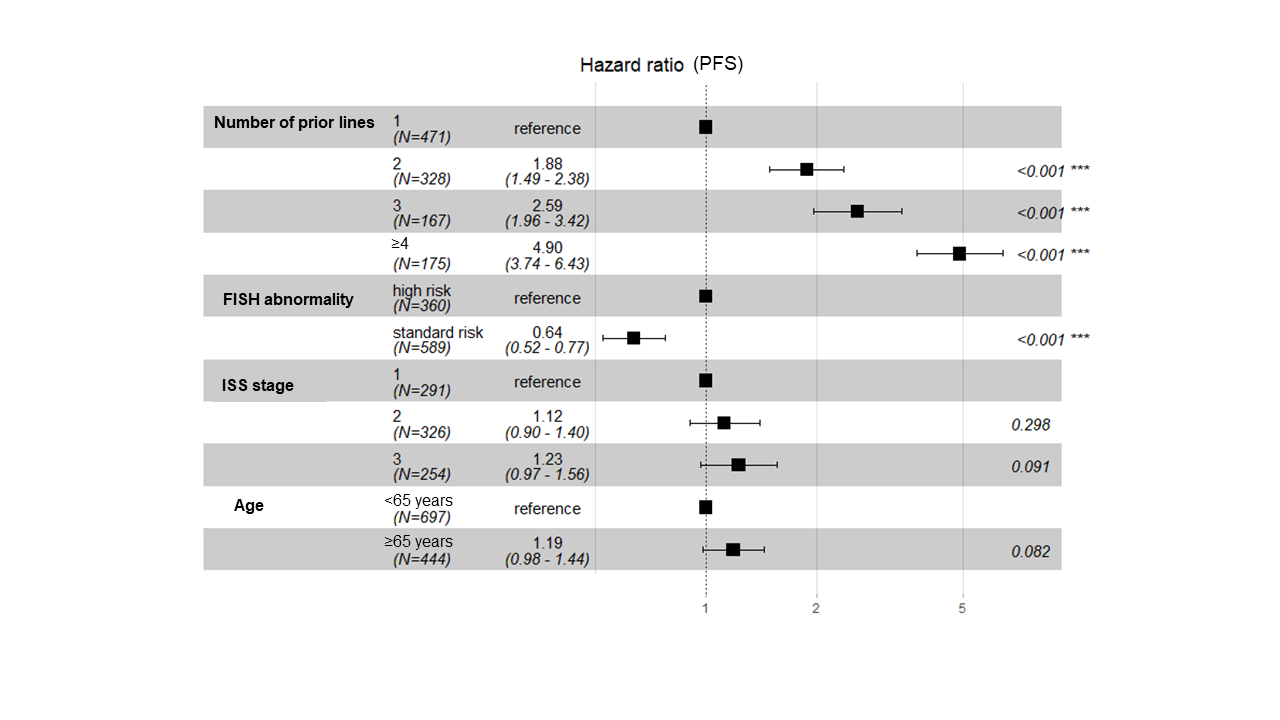
 **Supplementary Figure 3: Cox model hazard ratios for progression free survival (by lines of therapy)**

FISH indicates Fluorescence in-situ hybridization; ISS, International Staging System.


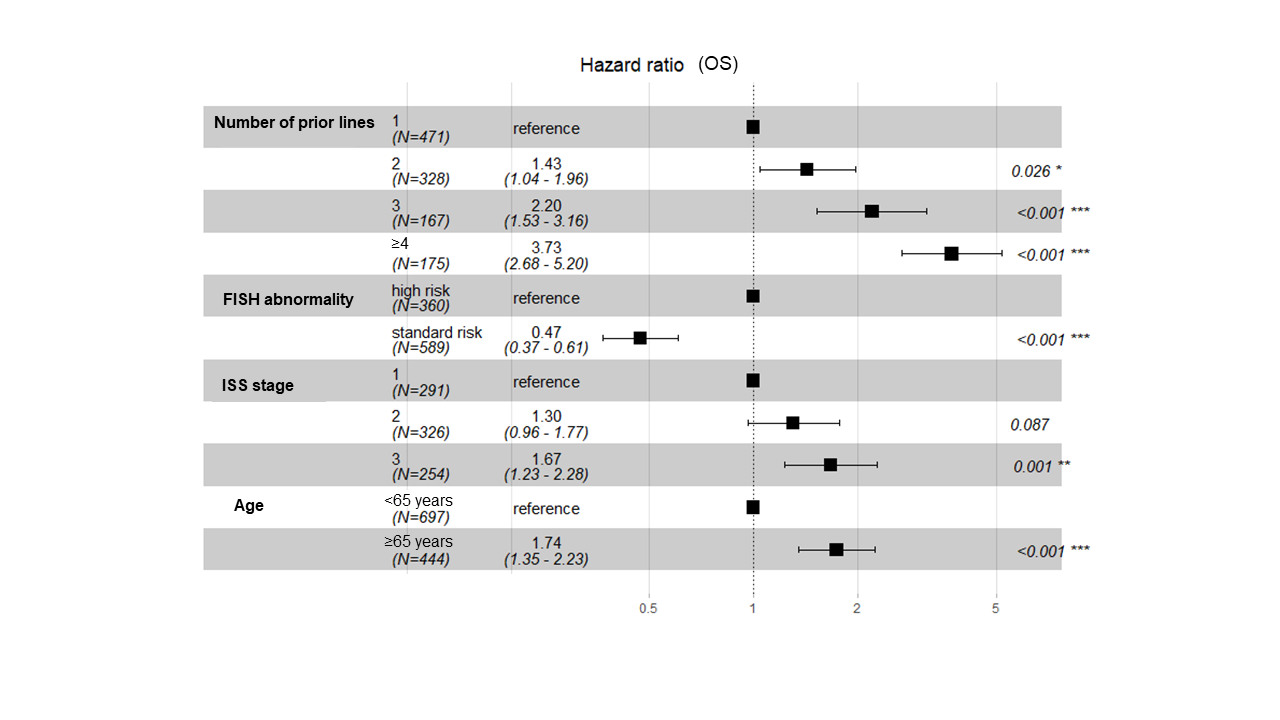
 **Supplementary Figure 4- Cox model hazard ratios for overall survival (by lines of therapy)**

FISH indicates Fluorescence in-situ hybridization; ISS, International Staging System.

**
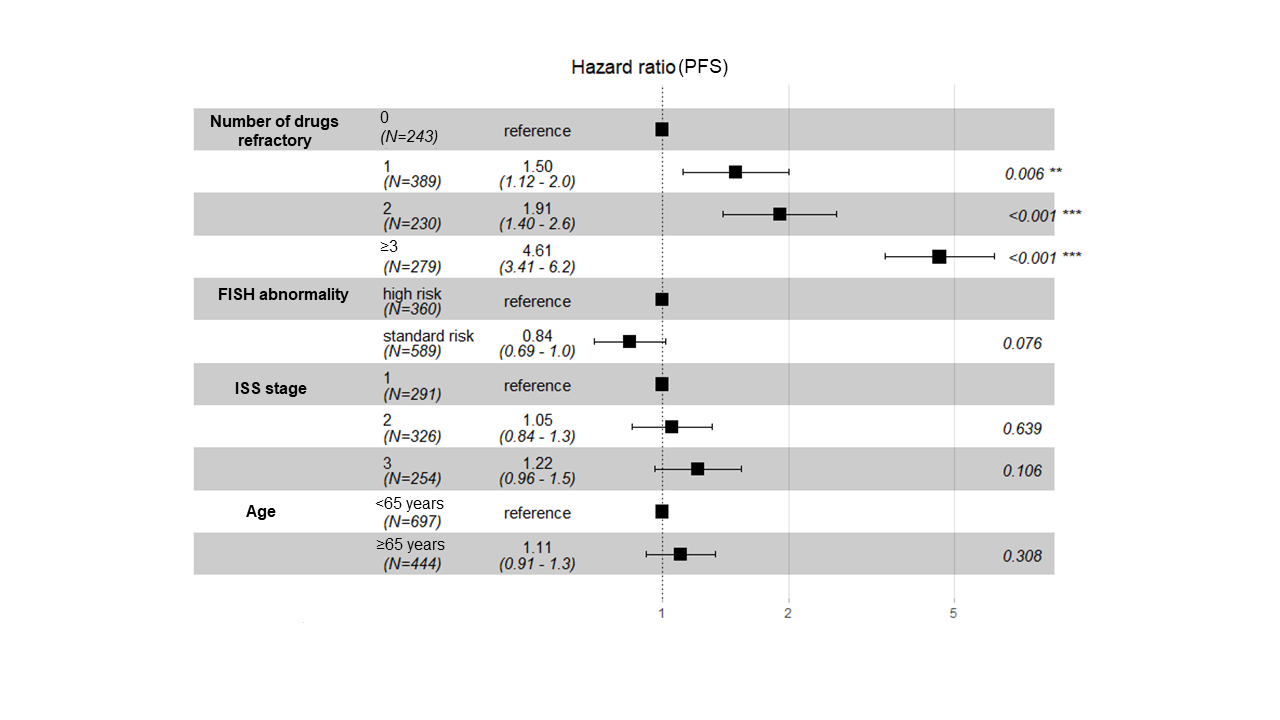
Supplementary Figure 5- Cox model hazard ratios for progression free survival (by refractoriness to number of drugs)**

FISH indicates Fluorescence in-situ hybridization; ISS, International Staging System.

**
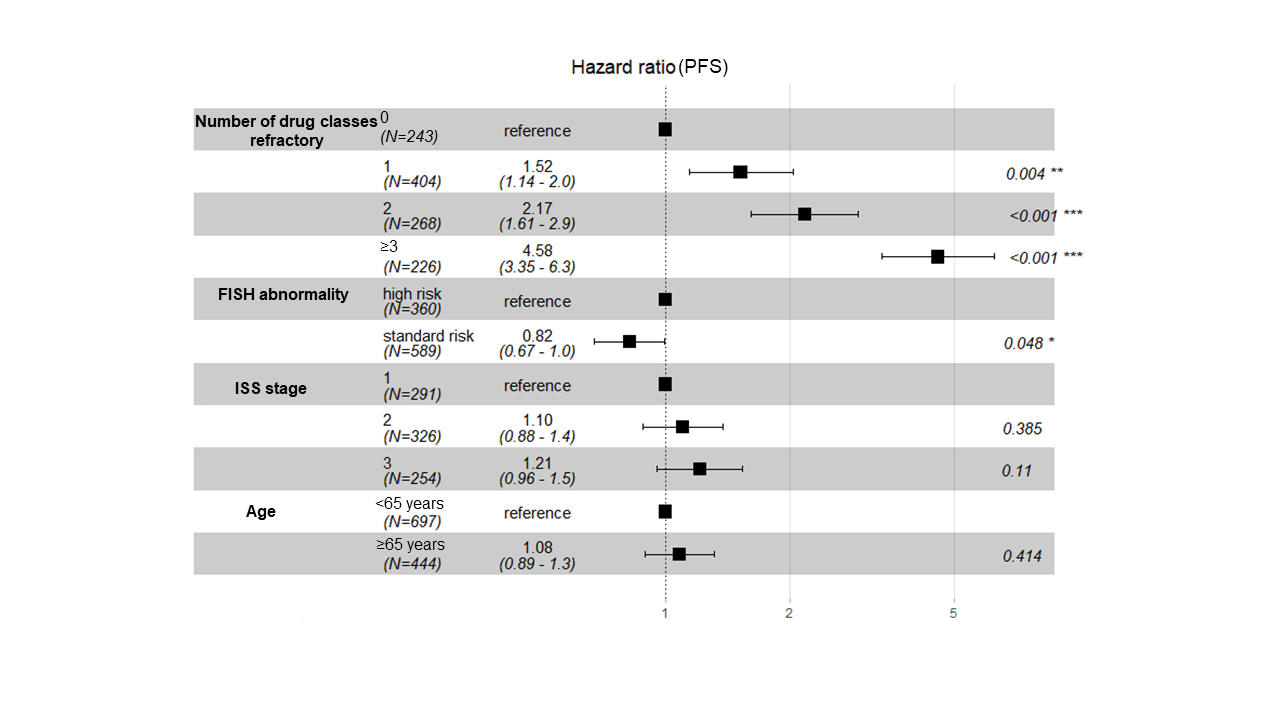

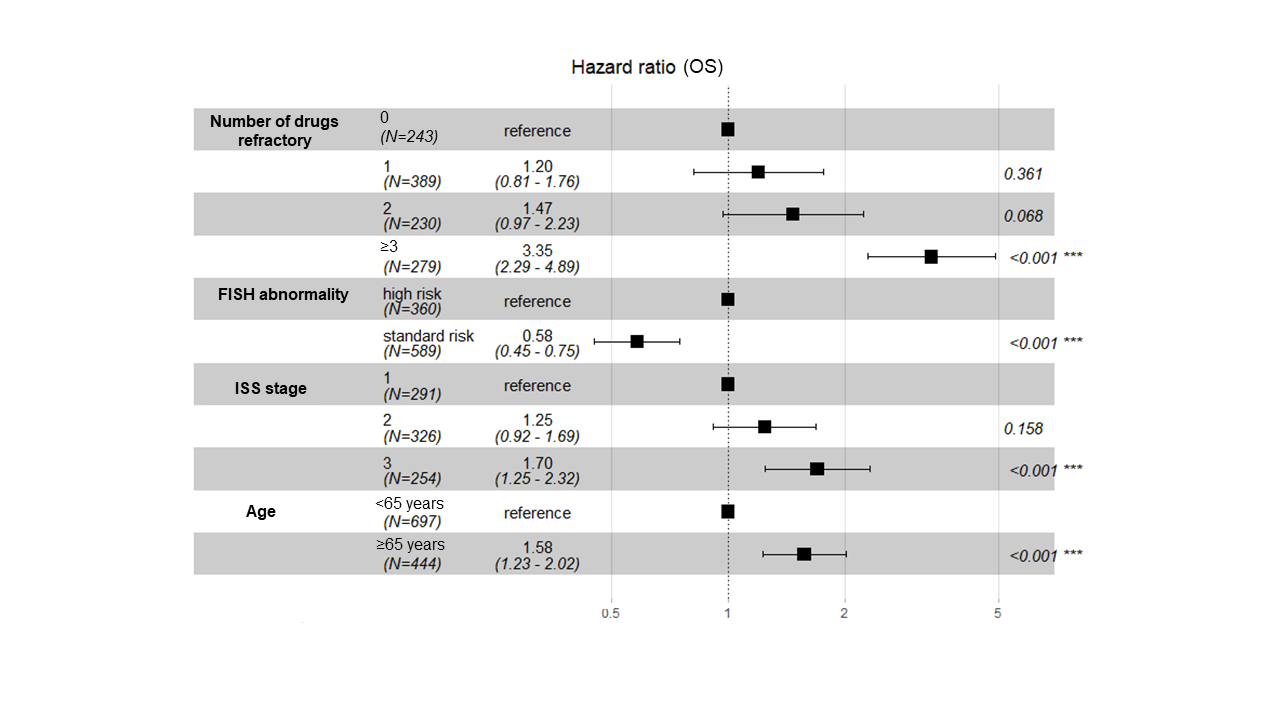
 Supplementary Figure 6- Cox model hazard ratios for overall survival (by refractoriness to number of drugs)**

FISH indicates Fluorescence in-situ hybridization; ISS, International Staging System.

**Supplementary Figure 7- Cox model hazard ratios for progression free survival (by refractoriness to number of drug classes)**

**
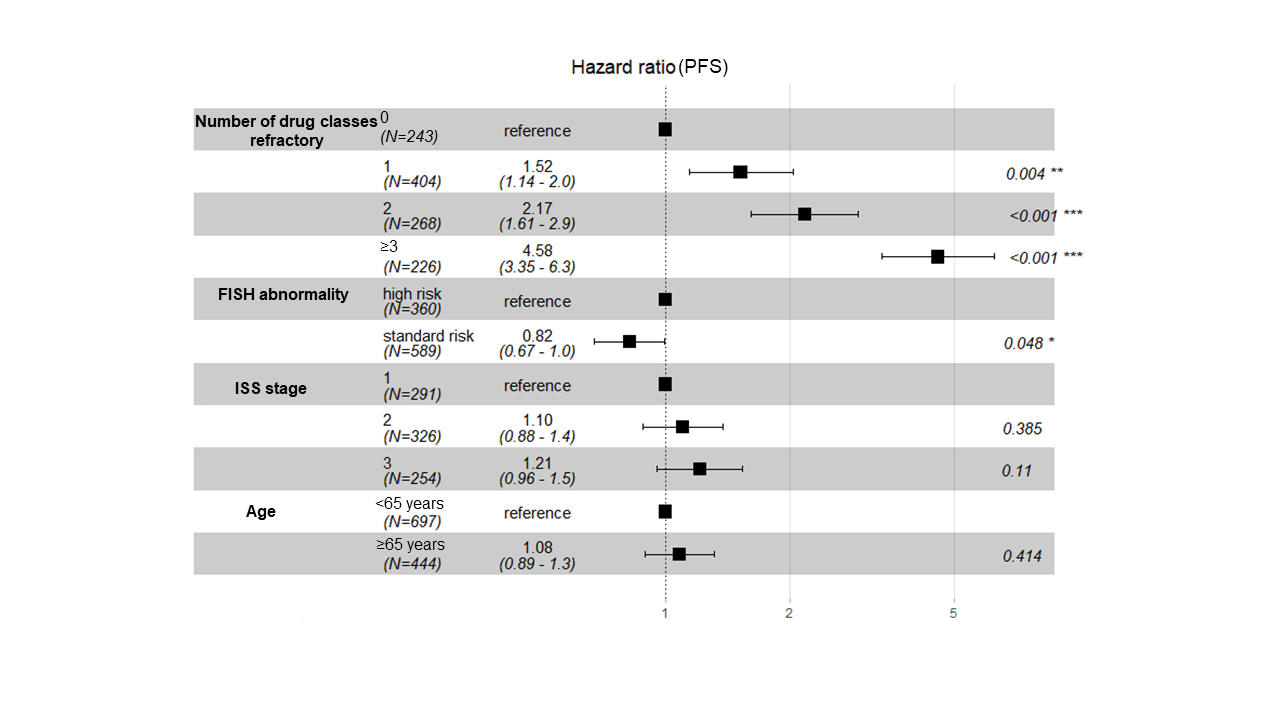
**

FISH indicates Fluorescence in-situ hybridization; ISS, International Staging System.

**
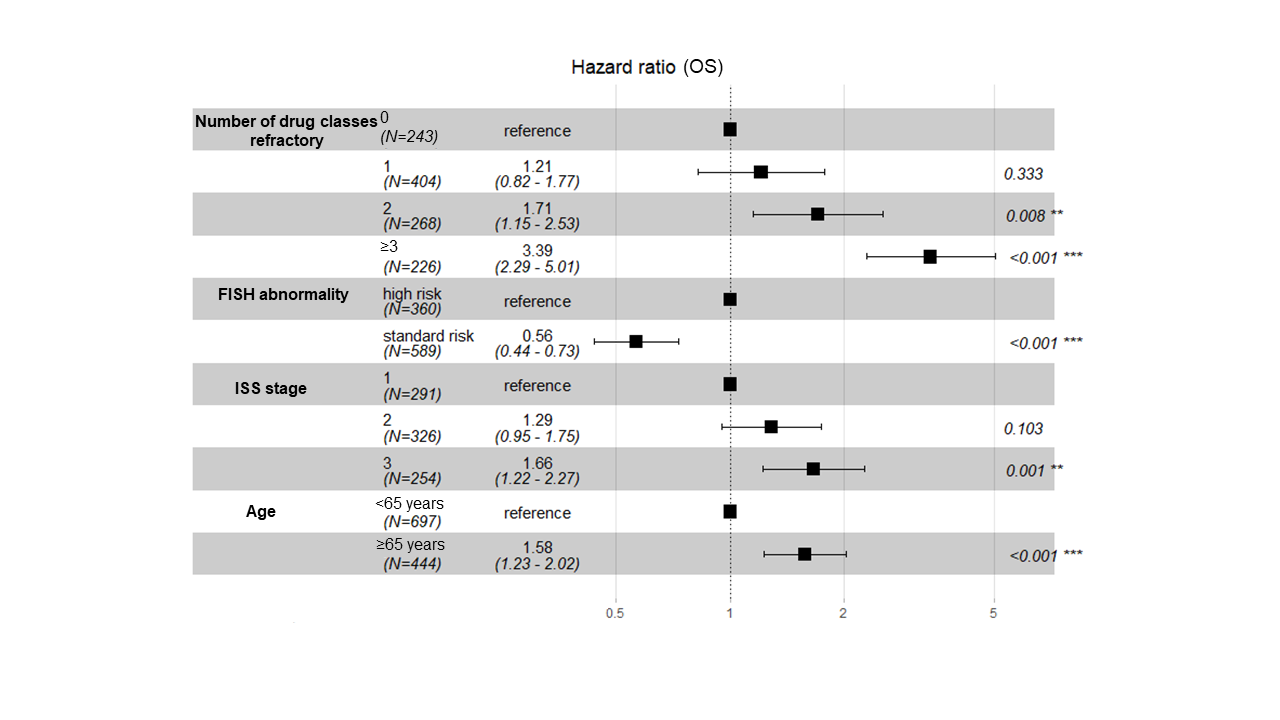
 Supplementary Figure 8- Cox model hazard ratios for overall survival (by refractoriness to number of drug classes)**

FISH indicates Fluorescence in-situ hybridization; ISS, International Staging System.

**Supplementary Figure 9A- Redistribution from lines of therapy to number of drugs refractory, Supplementary Figure 9B- Redistribution from lines of therapy to number of drug classes refractory.** Most of the redistribution was seen in patients who had received 2 and 3 prior lines of therapy. Classifications based on refractoriness to number of drugs vs number of drug classes were almost identical with only 68 (6%) patients migrating between the number of drugs they were refractory to, to the number of drug classes they were refractory to (redistribution figure not shown).


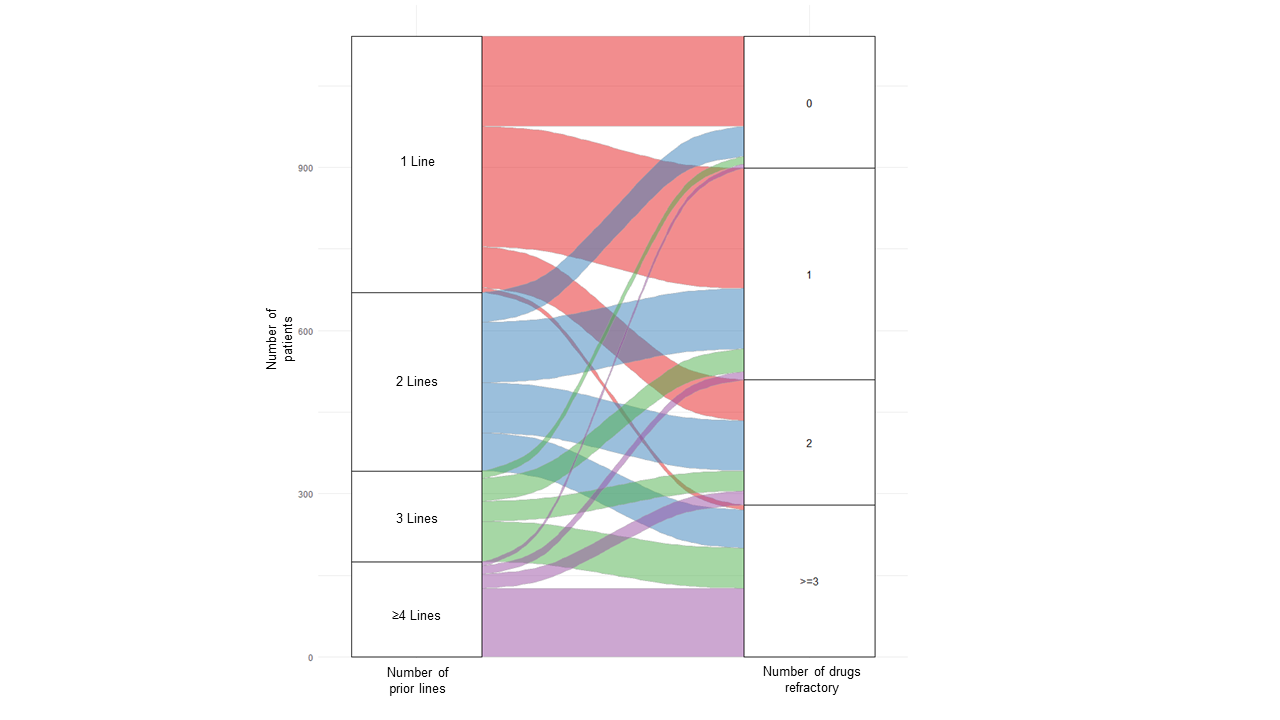

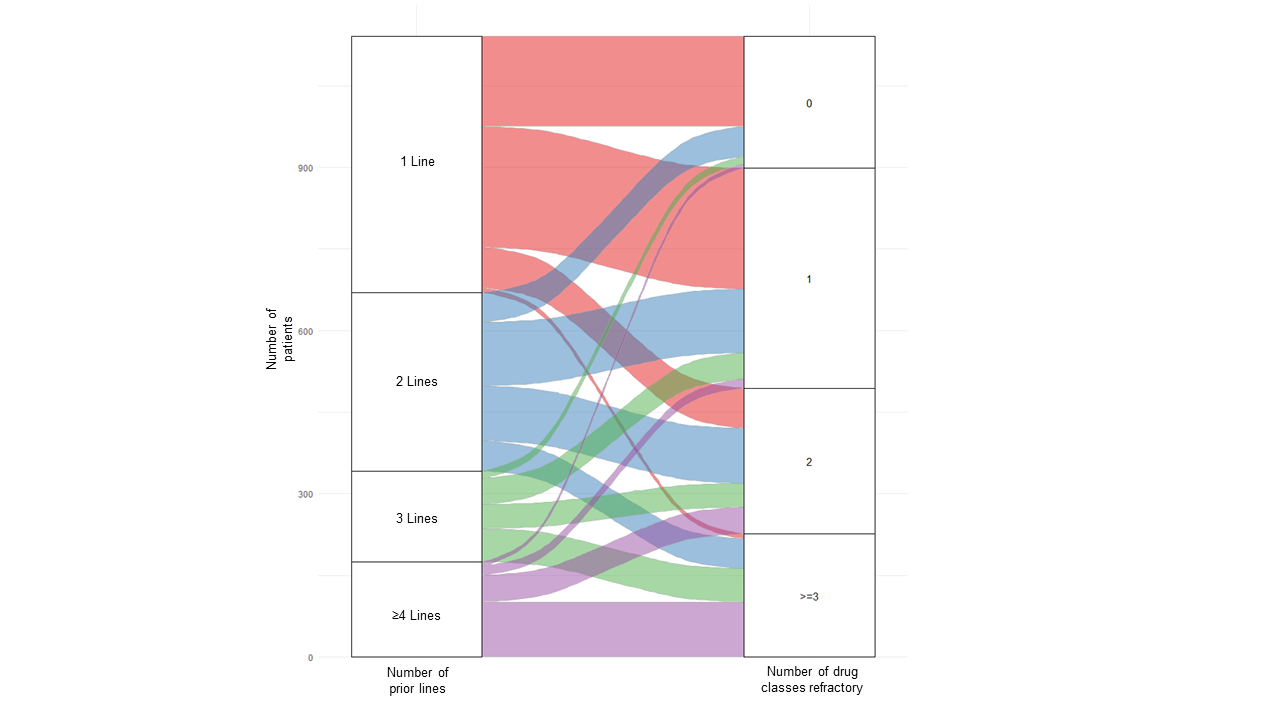


**Supplementary Figure 10A- Progression Free Survival (PFS) by refractoriness to number of drug classes (in patients that had received 1-3 prior lines of therapy) Supplementary Figure 10B- Overall Survival (OS) by refractoriness to number of drug classes (in patients that had received 1-3 prior lines of therapy).** The median PFS for not refractory patients was 41 months (95% CI: 31 – 56 months), for 1 class refractory was 23 months (95% CI: 20 – 28 months), for 2 class refractory was 13 months (95% CI: 11 – 18 months), and for >=3 class refractory was 8 months (95% CI: 6 – 11 months). The PFS for all these groups were significantly different from each other (p<0.001 for all pairs). The median OS for not refractory patients was not reached, for 1 class refractory was 65 months, for 2 class refractory was 64 months, and for >=3 class refractory was 31 months. The OS was not statistically different between 1 class refractory and 2 class refractory patients (p=0.16) and was statistically different among other groups (p=0.02 for 1 class refractory vs not refractory patients, p<0.01 for all other pairs)


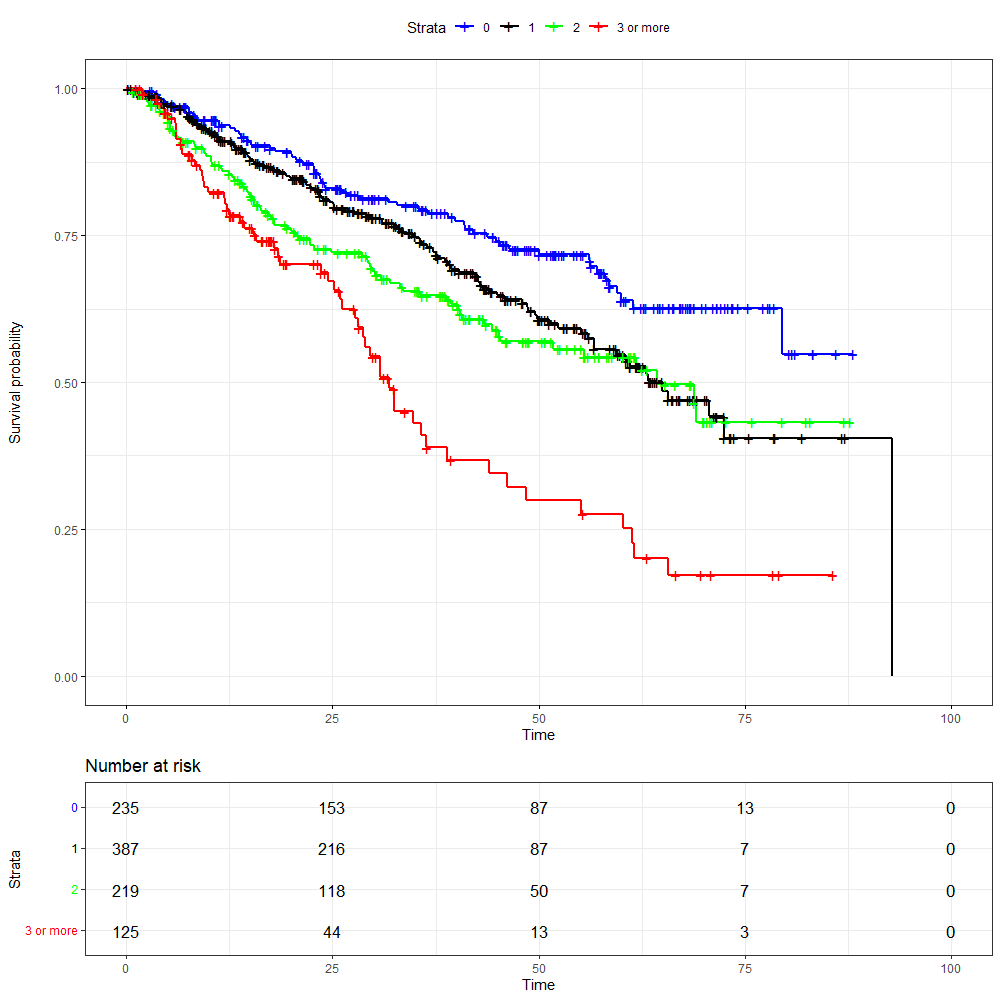

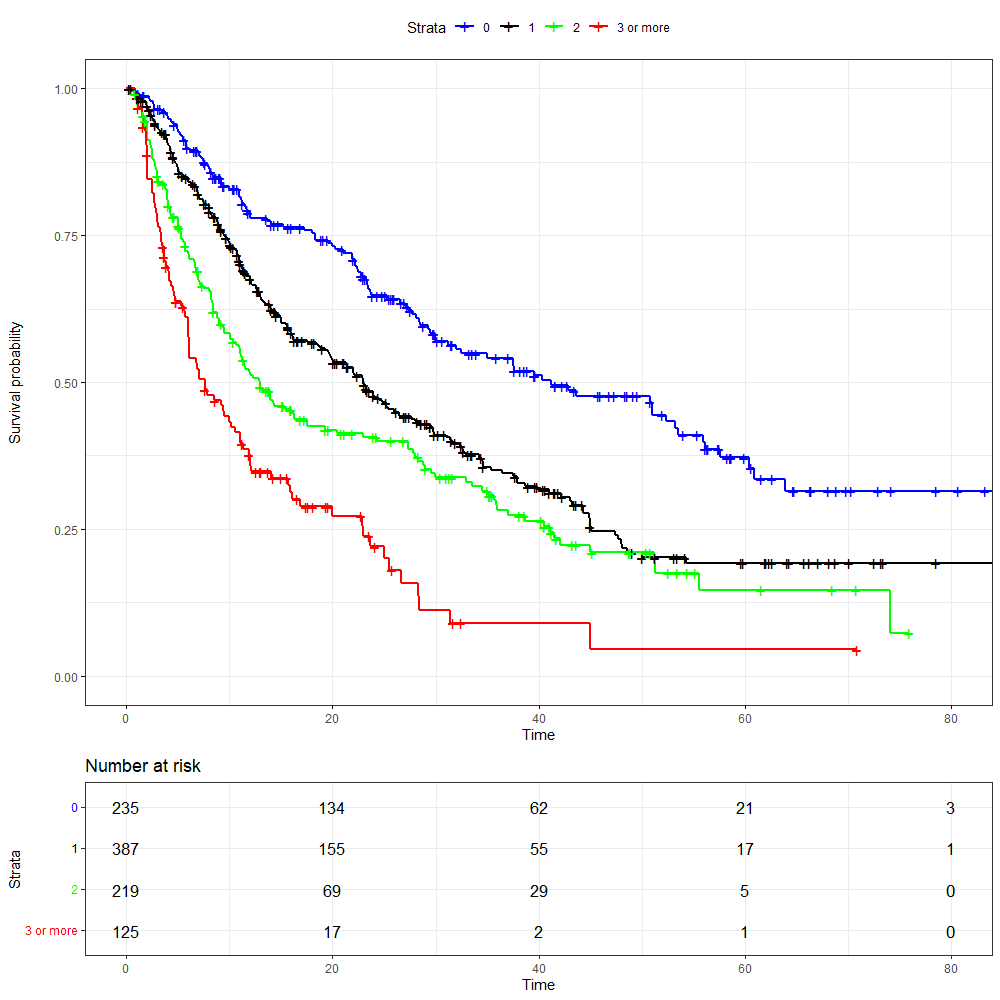


**Supplementary Figure 11A- Progression free survival by refractoriness to last line of therapy, Supplementary Figure 11B- Overall survival by refractoriness to last line.** Patients who were refractory to last line had a worse PFS as compared to patients who were not refractory to last line (median PFS 13 months vs 28 months, p<0.001). Patients who were refractory to last line of therapy had a worse OS from index relapse, as compared to patients not refractory to last line (median OS 51 months vs 80 months, p<0.001)

**
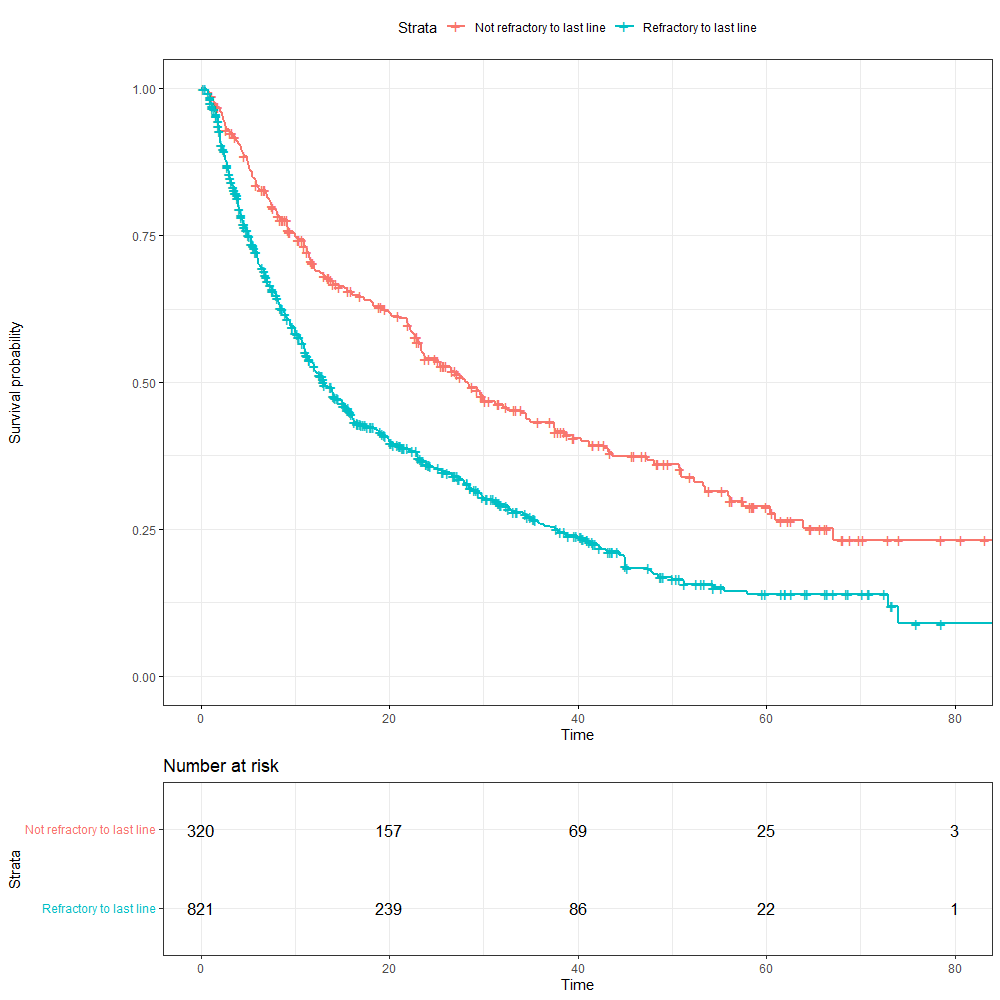

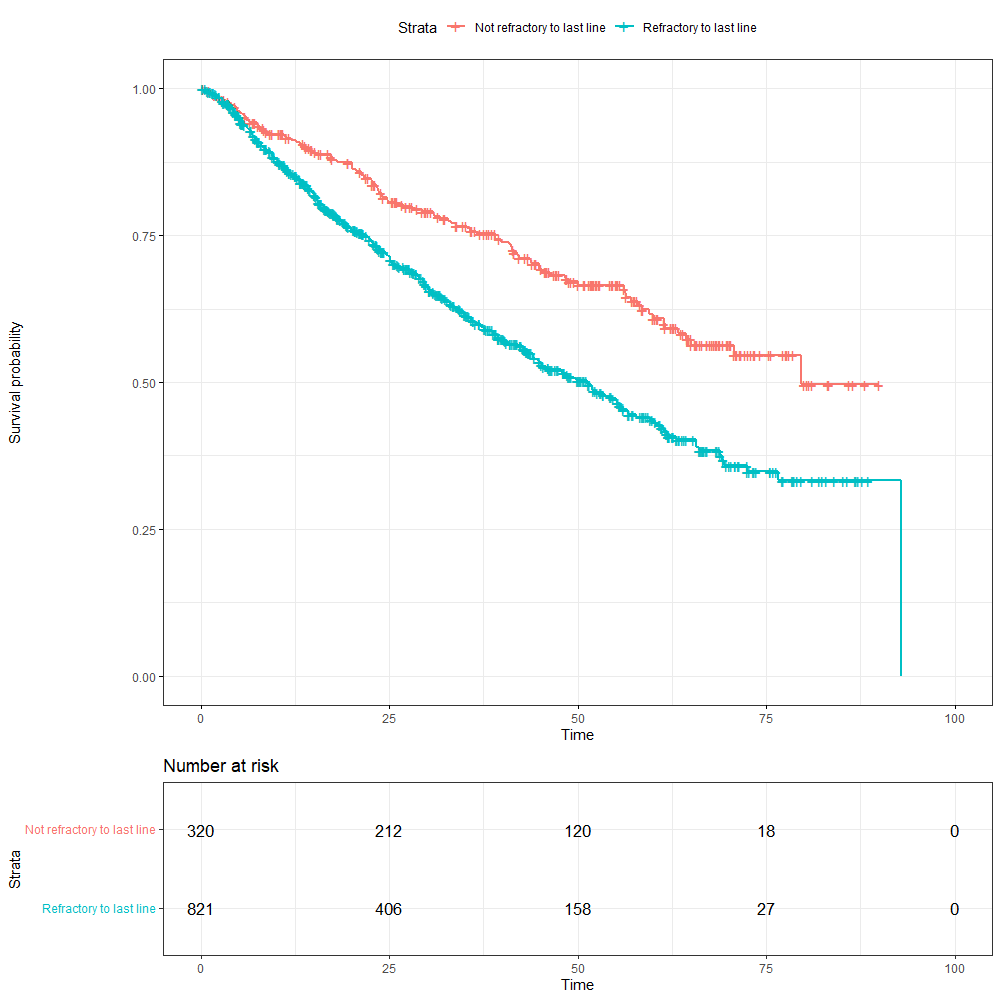
**

**Supplementary Figure 12A- Progression free survival by receipt of previously refractory to drug at index relapse, Supplementary Figure 12B- Overall survival by receipt of previously refractory to drug at index relapse.** The PFS for patients who received a drug at index relapse that they had been previously refractory to was significantly worse than patients who had received new/ not refractory to drugs (median PFS 11 months vs 21 months, p<0.001) There was no OS difference between these patient groups (median PFS 56 months vs 61 months, p=0.09)

**
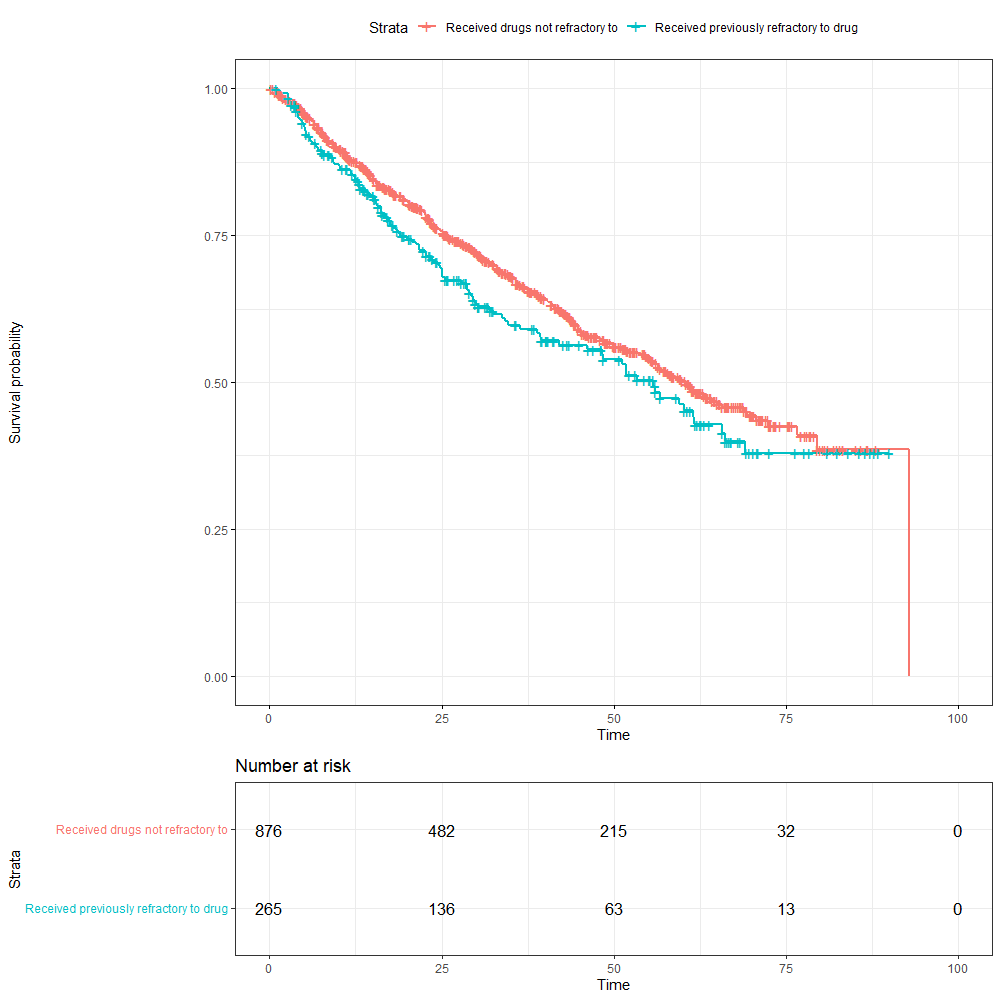

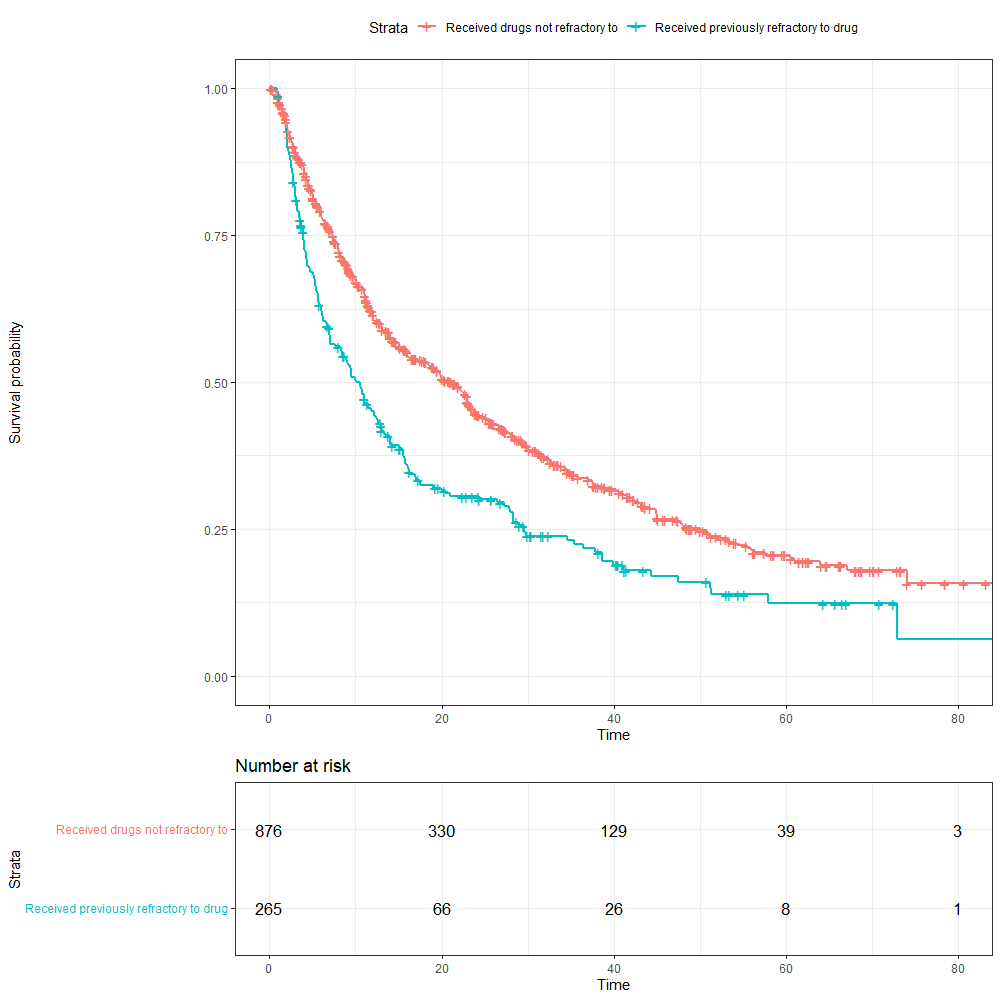
**
